# Severe Acute Pancreatitis Prediction: A Model Derived From a Prospective Registry Cohort

**DOI:** 10.7759/cureus.46809

**Published:** 2023-10-10

**Authors:** Juan Carlos Barrera Gutierrez, Ian Greenburg, Jimmy Shah, Priyanka Acharya, Mingyang Cui, Elaina Vivian, Brad Sellers, Prashant Kedia, Paul R Tarnasky

**Affiliations:** 1 Methodist Digestive Institute, Methodist Health System, Dallas, USA; 2 Gastroenterology Fellowship Program, Methodist Health System, Dallas, USA; 3 Clinical Research Institute, Methodist Health System, Dallas, USA; 4 Performance Improvement, Methodist Health System, Dallas, USA; 5 Emergency, Methodist Health System, Dallas, USA; 6 Gastroenterology, Methodist Health System, Dallas, USA

**Keywords:** severe acute pancreatitis, prognostication, early medical management, severity prediction, acute pancreatitis

## Abstract

Background

Severe acute pancreatitis (SAP) has a mortality rate as high as 40%. Early identification of SAP is required to appropriately triage and direct initial therapies. The purpose of this study was to develop a prognostic model that identifies patients at risk for developing SAP of patients managed according to a guideline-based standardized early medical management (EMM) protocol.

Methods

This single-center study included all patients diagnosed with acute pancreatitis (AP) and managed with the EMM protocol Methodist Acute Pancreatitis Protocol (MAPP) between April 2017 and September 2022. Classification and regression tree (CART®; Professional Extended Edition, version 8.0; Salford Systems, San Diego, CA), univariate, and logistic regression analyses were performed to develop a scoring system for AP severity prediction. The accuracy of the scoring system was measured by the area under the receiver operating characteristic curve.

Results

A total of 516 patients with mild (n=436) or moderately severe and severe (n=80) AP were analyzed. CART analysis identified the cutoff values: creatinine (CR) (1.15 mg/dL), white blood cells (WBC) (10.5 × 10^9^/L), procalcitonin (PCT) (0.155 ng/mL), and systemic inflammatory response system (SIRS). The prediction model was built with a multivariable logistic regression analysis, which identified CR, WBC, PCT, and SIRS as the main predictors of severity. When CR and only one other predictor value (WBC, PCT, or SIRS) met thresholds, then the probability of predicting SAP was >30%. The probability of predicting SAP was 72% (95%CI: 0.59-0.82) if all four of the main predictors were greater than the cutoff values.

Conclusions

Baseline laboratory cutoff values were identified and a logistic regression-based prognostic model was developed to identify patients treated with a standardized EMM who were at risk for SAP.

## Introduction

Acute pancreatitis (AP) is the third most common gastrointestinal reason for hospitalization in the United States and accounts for approximately 300,000 hospitalizations annually and nearly three billion dollars in direct healthcare costs [1]. The Revised Atlanta Classification (RAC) defines AP severity as mild, moderately severe, and severe, based on organ failure and complications [2]. Severe AP (SAP), defined as persistent organ failure (>48 hrs), is observed in 10%-20% of patients with AP. Overall mortality in AP patients is <10%; however, mortality is markedly increased (>30%) in those with persistent organ failure [3-5]. Mortality is much higher when organ failure of two more systems is apparent early (i.e., within the first week) [6]. 

Classification of final AP severity is necessary for benchmarking outcomes. It is more important, however, to identify at baseline those patients at risk for poor outcomes. Unfortunately, highly accurate predictive models to identify SAP in patients on presentation do not exist [7-9]. While clinical presentations of patients with early SAP are typically obvious, less than half will be graded as having SAP [10]. Conversely, the clinical manifestations of severity can often only become apparent after the first week [6,11].

Fortunately, most AP cases are mild and can be accurately identified as such on presentation [12]. Early prediction of SAP promotes patient triage to an appropriate level of care (e.g., ICU) and to direct early medical management (EMM; e.g., goal-directed fluid resuscitation) [13,14]. We have previously demonstrated that patients managed by the standardized EMM Methodist Acute Pancreatitis Protocol (MAPP) had significantly less SAP and a lower length of stay [14]. Prior AP severity scoring tools were derived from retrospective cohorts without standardized EMM so are subject to confounding variables. The objective of this study was to identify a prognostic model for AP severity based on prospective data from a standardized cohort treated according to an evidence-based EMM protocol.

## Materials and methods

Study design and patients

The MAPP was initiated in January 2015. MAPP is a multidisciplinary EMM quality initiative that implements computerized physician orders, physician/nursing education, and patient navigator-directed care to optimize adherence to AP published guidelines (APPG) [9]. Data from patients treated with MAPP are compiled prospectively in a registry.

Analysis in the current study included data from all patients in the registry diagnosed with AP from April 2017 to September 2022 who were managed per the MAPP. Patients were included in the analysis if they met APPG diagnostic criteria: pain consistent with AP, serum amylase and/or lipase >3X the upper limit of normal, and/or characteristic imaging findings. Exclusion criteria included age <18 years, transfers from outside hospitals, post-endoscopic retrograde cholangiopancreatography AP, and trauma-related AP.

The study was approved by the WCG Institutional Review Board and was found to meet the requirements for a waiver of consent under 45 CFR 46 116(f) (2018 Requirements).

Data collection and outcomes

Baseline data (from admission in the emergency department) and measured outcomes included age, body mass index (BMI), white blood cell (WBC) count, hematocrit (HCT), creatinine (CR), C-reactive protein (CRP), procalcitonin (PCT), systemic inflammatory response syndrome (SIRS), presence of pleural effusion on imaging, bedside index of severity in acute pancreatitis (BISAP) score [15], and harmless AP score (HAPS) [16]. The HAPS criteria are met if all three of the following conditions are present: absent peritoneal exam findings, normal CR, and normal HCT. Systemic complications within 48 hrs were noted including need for mechanical ventilation, hypotension with a systolic blood pressure (SBP) <90 mmHg, need for vasopressor support, altered mental status, cardiovascular failure, renal failure, and/or organ failure. Whether or not patients underwent computed tomography (CT) imaging was also recorded. Severity outcomes were defined as mild, moderate, and severe as per the RAC [2]. The final AP severity for data analyses was categorized as either mild or severe; the severe category included severe and moderately severe.

Statistical analysis

All statistical analyses were carried out using SAS on Demand for Academics (SAS Institute Inc., Cary, NC) and Minitab Statistical Software (version 21.1.0; Minitab, LLC, State College, PA). Normally distributed variables are presented as means and standard deviations (SD). Non-normally distributed variables are presented as medians and minimum and maximum values. These variables were compared using t-tests or Wilcoxon tests, as appropriate. Categorical variables were summarized with percentages and compared using Pearson's chi-square test.

The cutoff values of continuous variables were established using classification and regression tree (CART®) analysis (CART® Professional Extended Edition, version 8.0; Salford Systems, San Diego, CA), providing equal probability for mild and SAP, and obtaining the highest sensitivity and specificity of each cutoff point. The cutoff values were used to convert continuous variables into binary variables. When the value of the predictor was lower than or equal to the cutoff value, it was assigned a value of zero. If the value of the predictor was greater than the cutoff value, it was assigned a value of one.

CART analysis utilizes two basic principles, classification and regression, to formulate a simple output that can be easily interpreted. Classification trees are used when the dependent variable is categorical. Regression trees are used when the dependent variable is continuous. The classification tree consists of categorical target variables, and the tree identifies the “class” into which the target variable would best fit. The optimal tree was selected according to its predictive accuracy and clinical relevance. The class probability function was used as the splitting rule for tree building: the node would not be split if the sample number was <30, and the terminal node would not be split if the sample number was <10. We used a 10-fold cross-validation method for validating the tree model, the overall performance of which was validated in the test sample using the area under the receiver operating characteristic curve. The CART analysis was also used to create a model that was compared to a logistic regression model.

Separately, logistic regression analysis was used to establish the association between baseline predictors and the severity of AP. A multivariable logistic regression model was constructed with backward variable selection, including those variables that were not highly correlated according to Pearson’s correlation analysis to avoid multicollinearity. The concordance (c) statistic and Hosmer-Lemeshow test were used to validate and calibrate the model. Additional models with two and three variables were included and reported with the c statistics. Missing values were handled with listwise deletion, and a p value<0.05 was considered statistically significant.

## Results

As shown in Table 1, 516 patients were included in this prospective study, of which 436 (84.5%) had mild AP and 80 (15.5%) had SAP. The mean age of patients was 49.4 years with 51.9% females and 48.1% males. The most common etiology of AP in our cohort was biliary (36%) and alcohol-induced (28%). The proportion of patients who met the definition for potential "not harmless" AP (i.e., they did not meet HAPS criteria) [16] was significantly higher among those with SAP compared to mild AP (73% vs. 51%; p=0.0004). Of the patients with SAP, 64% met the SIRS criteria. The percentage of patients with BISAP scores ≥2 was higher among those with SAP compared to those with mild AP (39% vs. 13%; p≤0.0001). Age, baseline CR, HCT, WBC count, CRP, PCT, and blood urea nitrogen (BUN) levels were significantly higher in patients with SAP compared to those with mild AP (all p values <0.05).

**Table 1:** Baseline characteristics of the sample population stratified by pancreatitis severity *Variables non-normal distributed Abbreviations: BISAP, bedside index of severity in acute pancreatitis; BMI, body mass index; BUN, blood urea nitrogen; CR, creatinine; CRP, c-reactive protein; EtOH, ethyl alcohol; HAPS, harmless acute pancreatitis score; HCT, hematocrit; HTG, hypertriglyceridemia; PCT, procalcitonin; SIRS, systemic inflammatory response system; WBC, white blood cell

Variable	Overall (N=516)	Mild pancreatitis (N=436)	Severe pancreatitis (N=80)	p value
Age (years), mean (SD)	49.4 (17.6)	48.5 (17.1)	54 (19.5)	0.01
BMI, (kg/m^2^), mean (SD)	29.9 (7.6)	26.6 (7.8)	29.8 (6.6)	0.9
Gender, n (%)				
Female	268 (52%)	240 (55%)	28 (35%)	0.001
Male	248 (48%)	196 (45%)	52 (65%)	
Etiology, n (%)				
Biliary	188 (36%)	166 (38%)	22 (28%)	0.0004
EtOH	143 (28%)	124 (29%)	19 (24%)	
HTG	32 (6%)	19 (4%)	13 (16%)	
Unknown	153 (30%)	127 (29%)	26 (17%)	
HAPS, n (%)				
Not harmless	280 (54%)	222 (51%)	58 (73%)	0.0004
Harmless	236 (46%)	214 (49%)	22 (27%)	
SIRS, n (%)				
No	331 (64%)	302 (70%)	29 (36%)	<0.0001
Yes	185 (36%)	134 (30%)	51 (64%)	
BISAP, n (%)				
0	208 (40%)	200 (46%)	8 (10%)	<0.0001
1	222 (43%)	181 (42%)	41 (52%)	
2	68 (13%)	48 (11%)	20 (25%)	
3	13 (3%)	7 (1%)	6 (8%)	
4	5 (1%)	0 (0%)	5 (6%)	
Lab values, mean (SD)				
HCT (%)	40.8 (6.0)	40.5 (5.9)	42.6 (6.6)	0.005
CR (mg/dL)	0.8 (0.6-6.2)	0.7 (0.3-3.2)	1.1 (0.3-6.2)	<0.0001*
WBC (x 10^9^ WBCs/L)	10.5 (2.4-3.1)	10 (2.4-31)	13.2 (4.4-28)	<0.0001*
CRP (mg/dL)	47 (13-380)	12 (5-359)	21 (5-380)	<0.0001*
PCT (ng/mL)	0.08 (0.03-66)	0.07 (0.3-51)	0.19 (0.04-65)	0.01*
BUN (mg/dL)	13 (2-18)	13 (2-18)	17 (2-82)	0.001*

Table 2 shows the results of logistic regression covariates versus the severity of pancreatitis. All the variables tested, except BMI (no patients with a BMI<19.15 kg/m^2^ developed SAP during the study period), showed a significantly increased risk of SAP (p<0.05). Patients with hypertriglyceridemia had 3.8 times the risk of SAP compared to other etiologies (95%CI: 1.8-8.2; p=0.0007).

**Table 2:** Logistic regression covariates versus severity of pancreatitis Abbreviations: BISAP, bedside index of severity in acute pancreatitis; BMI, body mass index; BUN, blood urea nitrogen; CI, confidence interval; CR, creatinine; CRP, c-reactive protein; HAPS, harmless acute pancreatitis score; HCT, hematocrit; HTG, hypertriglyceridemia; OR, odds ratio; PCT, procalcitonin; SIRS, systemic inflammatory response system; WBC, white blood cell

Variable	OR	Coeff	95%CI	p value
Age	1.02	0.0174	1.04-1.03	0.012
Gender				
Female	Ref			
Male	2	0.822	1.4-3.7	0.001
Etiology				
HTG	3.8	1.339	1.8-8.2	0.0007
Other	Ref			
HAPS				
Not harmless	2.5	0.9327	1.5-4.3	0.0005
Harmless	Ref			
BISAP in 2 categories				
0	Ref			
≥ 1	7.6	2.032	3.6-16	0.0001
SIRS				
No	Ref			
Yes	4	1.377	2.4-6.5	0.0001
Age (years)				
≤ 63.5	Ref			
> 63.5	2.6	0.937	1.5-4.3	0.0001
BMI (*kg/m^2^*)				
≤ 19.15	Ref			
> 19.15	100	NA	NA	NA
CR (mg/dL)				
≤ 1.15	Ref			
> 1.15	8.7	2.16	5-14.8	0.0001
HCT (%)				
≤ 49.2	Ref			
> 49.2	3.6	1.295	1.9-7	0.0001
WBC (x 10^9^ WBCs/L)				
≤ 11	Ref			
> 11	3.3	1.2	2-5.6	0.0001
CRP (mg/dL)				
≤ 45.5	Ref			
> 45.5	3.7	1.373	2.1-6.5	0.0001
PCT (ng/mL)				
≤ 0.155	Ref			
> 0.155	3.9	1.373	2.3-6.5	0.0001
BUN (mg/dL)				
≤ 37.5	Ref			
> 37.5	22	3.1	8.6-58	0.0001

Cutoff values and CART analysis

**Figure 1:**
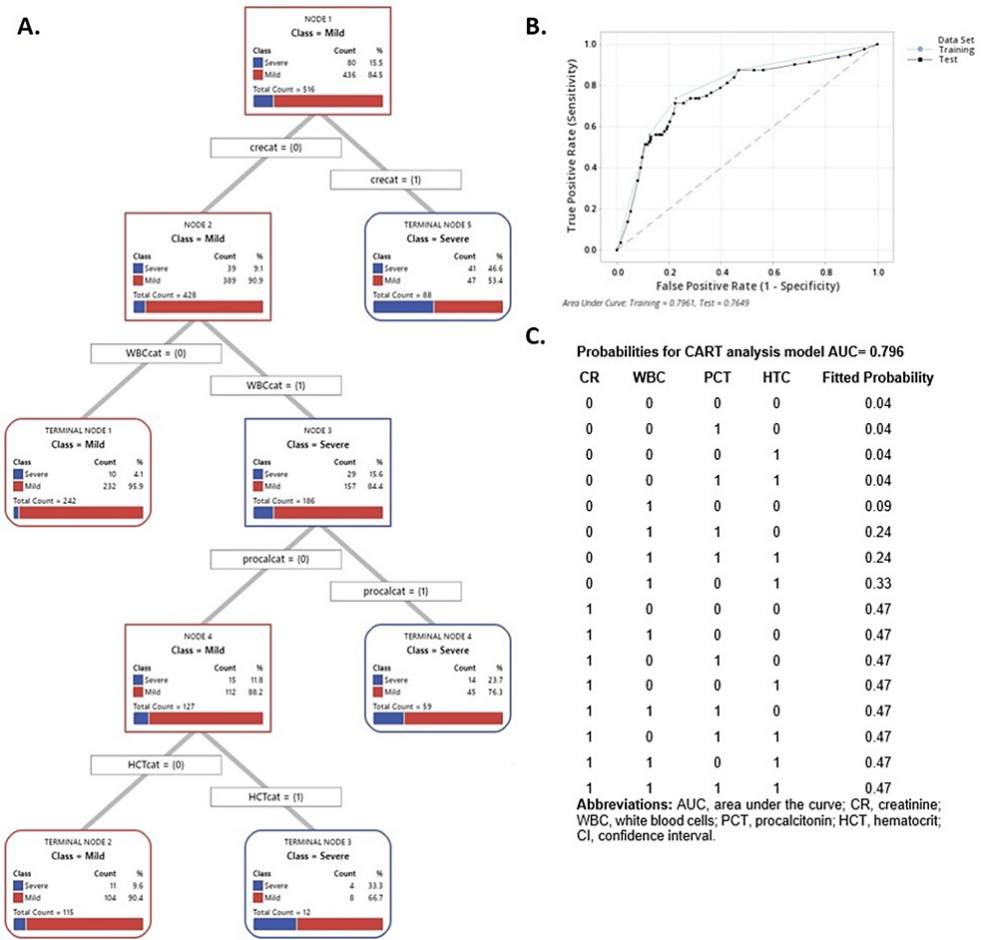
Classification tree for the severity of acute pancreatitis with four variables creatinine, white blood cells, procalcitonin, and hematocrit. (A) Alternative tree diagram. (B) Area under the curve (AUC) for classification tree for severity of acute pancreatitis with four variables creatinine, white blood cells, procalcitonin and hematocrit. (C) Probabilities for the CART analysis model (AUC=0.796)

The predictor variable cutoff values, sensitivity, specificity, and area under the curve (AUC) are found in Table 3. The variables included in the CART analysis (i.e., age, BMI, CR, CRP, PCT, HCT, BUN, SIR, and HAPS) were all categorized into two levels. When the value of the predictor was lower than or equal to the cutoff value, it was assigned a value of zero. If the value of the predictor was greater than the cutoff value, it was assigned a value of one. The CART analysis identified four main variables to satisfy the classification of severe and mild pancreatitis: CR, WBC, PCT, and HCT. The decision tree with the lower misclassification cost (0.51%) and its corresponding AUC (AUC=0.796) are found in Figures 1A-1B, respectively. The CART model produced five terminal nodes with a fitted constant probability of severity of AP. The sensitivity of the CART model was 0.738, and the specificity was 0.773.

**Table 3:** Cutoff values sensitivity, specificity, and area under the curve * No patients with BMI<19.15 kg/m2 had severe acute pancreatitis Abbreviations: AUC, area under the curve; BMI, body mass index; BUN, blood urea nitrogen; CR, creatinine; CRP, c-reactive protein; HCT, hematocrit; PCT, procalcitonin; WBC, white blood cell

Variable	Cutoff	Sensitivity	Specificity	AUC
Age (years)	> 63.5	37.5	81	0.59
BMI (kg/m^2^)	> 19.15	100	NA*	0.53
CR (mg/dL)	> 1.15	43.6	82.1	0.66
HCT (%)	> 49.2	22	94	0.57
WBC (x 10^9^ WBCs/L)	> 11	74.5	59	0.67
CRP (mg/dL)	> 45.5	29	80	0.59
PCT (ng/mL)	> 0.155	54.5	73.2	0.64
BUN (mg/dL)	> 37.5	24	99.7	0.61

Patients with a CR level >1.15 mg/dL had a 47% probability of having SAP, regardless of the value of the other variables included in the model (Figure 1C). The subgroup of patients with a CR level ≤1.15 mg/dL, WBC >11 x 109/L, and PCT >0.155 ng/mL had a 24% probability of developing SAP. The subgroup of patients in the terminal node with CR ≤1.15 mg/dL and WBC ≤11 x 109/L had a 4% probability of developing SAP. The terminal node with patients with a CR level ≤1.15 mg/dL, WBC >11 x 109/L, and HCT >49.2% had a 33.3% probability of developing SAP, while patients in the same level but with a HCT ≤49.2% had only 10% probability. The CART analysis provided a fixed probability when the CR cutoff value is >1.15 mg/dL.

Logistic regression

**Figure 2:**
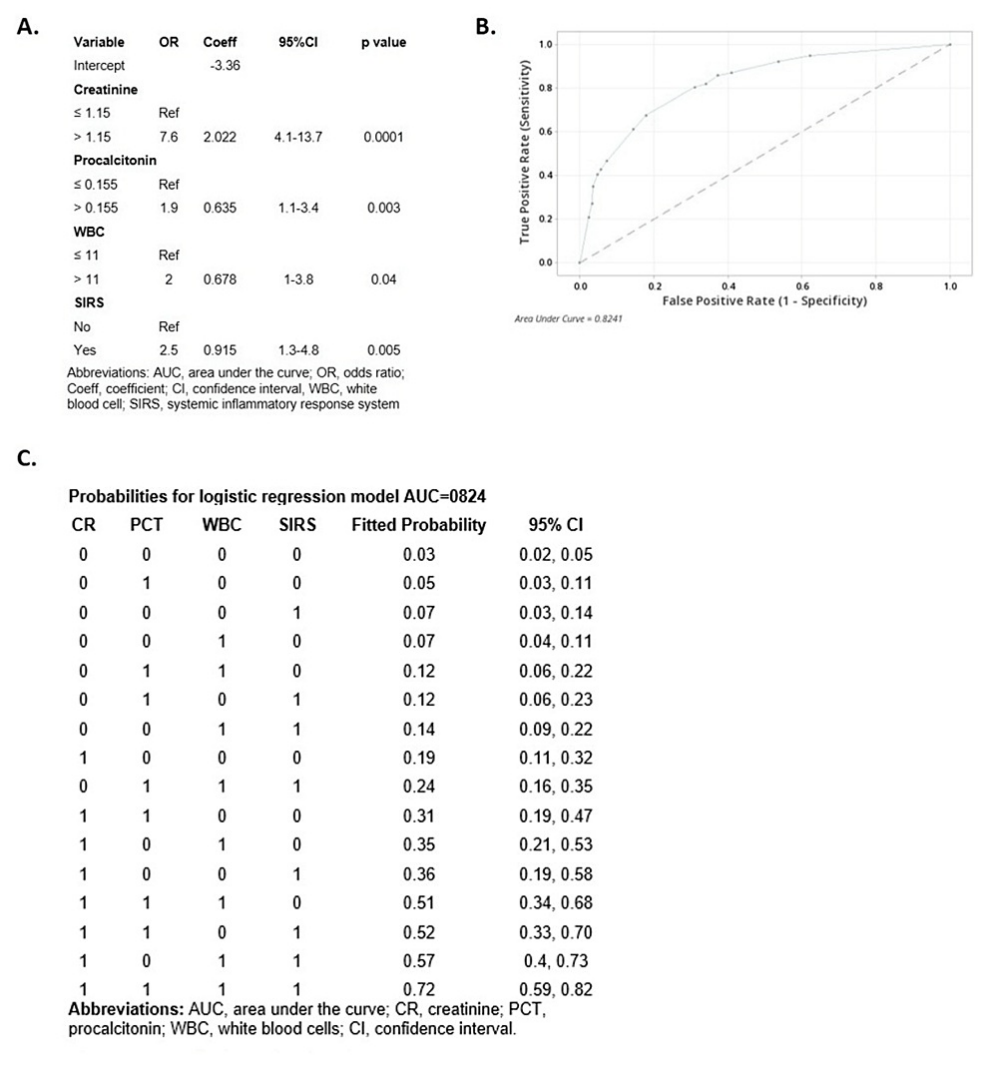
Multivariable logistic regression analysis for the prediction of severity of acute pancreatitis including the variables creatinine, white blood cells, procalcitonin, and systemic inflammatory response system. (A) Multivariable logistic regression model (AUC=0.824). (B) Receiver operating characteristic curve. (C) Probabilities for the logistic regression model

The association between demographic characteristics, baseline predictors (categorized into zero and one, based on the cutoff values), and AP severity was assessed by logistic regression analysis (Figure 2A). No patients with a BMI <19.15 kg/m^2^ developed SAP during the study period. In this bivariate analysis, all predictors with values exceeding their respective established cutoff values were significantly associated with SAP.

In the multivariate logistic analysis, BISAP and HAPS were excluded to avoid redundancy with SIRS, CR, and HCT. After adjustment for the covariates included in the model, CR (OR: 7.6, 95%CI: 4.1-13.7, p=0.0001), PCT (OR: 1.9, 95%CI: 1.1-3.4, p=0.003), WBC (OR: 2, 95%CI: 1-38, p=0.04), and SIRS (OR: 2.5, 95%CI: 1.3-4.8, p=0.005) were the main predictors of severity. The analysis showed that having a CR >1.15 mg/dL increased the probability of SAP by 7.5 times compared to a CR ≤1.15 mg/dL when the other covariates are held constant. Similar interpretations can be made for the other predictors. The AUC was 0.824 (Figure 2B), and the p value for the Hosmer-Lemeshow test was 0.824. This logistic model had a sensitivity of 0.787 and a specificity of 0.688.

The formula for the model is as follows:

Probability of severity (SAP) = exp(Y’)/(1=exp(Y’)

Y’ = -3.368 + 0.0 CR_0 + 2.022 CR_1 + 0.0 PCT_0 + 0.635 PCT_1 + 0.0 WBC_0 + 0.678 WBC_1 + 0.0 SIRS_0 + 0.915 SIRS_1

Figure 2C provides the probabilities of SAP according to the logistic regression model. The logistic regression model generated a probability that depended on each cutoff value of every predictor. While the CART analysis did not provide 95% CIs for the probabilities estimated (Figure 1C), the logistic regression model did.

Additional logistic regression models were constructed including only two variables, with CR (>1.15 mg/dL) always as one of the variables, for example, CR+PCT (>0.155 ng/mL), CR+WBC (>11 x 109/L), or CR+SIRS. These models all showed an AUC >71%. Table 4 shows that, when both predictors have values greater than their cutoff points, the probability of SAP was >50%.

**Table 4:** Probabilities for additional logistic regression models with two variables, where one is creatinine Abbreviations: AUC, area under the curve; CI, confidence interval; CR, creatinine; FP, fitted probability; PCT, procalcitonin; SIRS, systemic inflammatory response system; WBC, white blood cell

Probabilities for CR + PCT logistic regression model, AUC=0.71				Probabilities for CR + WBC logistic regression model, AUC=0.78				Probabilities for CR + SIRS logistic regression model, AUC=0.78			
CR	PCT	FP	95%CI	CR	WBC	FP	95%CI	CR	SIRS	FP	95%CI
0	0	0.06	0.04-0.09	0	0	0.05	0.03-0.08	0	0	0.05	0.03-0.08
1	0	0.34	0.25-0.47	1	0	0.31	0.21-0.44	1	0	0.31	0.21-0.43
0	1	0.15	0.1-0.22	0	1	0.14	0.1-0.2	0	1	0.17	0.1-0.23
1	1	0.57	0.44-0.68	1	1	0.58	0.46-0.7	1	1	0.63	0.5-0.74

## Discussion

While the incidence of AP, including outpatient encounters [17], may be decreasing, hospital admissions are increasing. An effective method exists for predicting mild AP attacks that may not need higher levels of care [12,16]. However, an accurate tool to predict SAP is required to initiate appropriate initial medical management, properly triage the level of care, and/or identify those that might benefit from transfer to another institution for a higher level of care [13,18,19]. An ideal predictive model would have a high sensitivity and a high negative predictive value. A highly sensitive model would optimally identify those at risk; we considered that a 30% probability of developing SAP was clinically relevant. A high negative predictive value would suggest minimal risk for SAP when the predictive score suggests a mild severity outcome.

We found that four objective baseline variables can predict the likelihood of developing SAP. The probability of developing SAP was <5% if none of the four baseline parameters were met, but as high as nearly 75% if all four baseline parameters were met. When baseline CR was >1.15 mg/dL and any one of the other baseline parameters (i.e., WBC>11 x 109 WBCs/L, PCT>45.5 ng/mL, or meeting SIRS criteria) were met, the probability of SAP was at least 30%. The probability for SAP was 20% when only the CR threshold was met, and <10% when only any of either WBC, PCT, or SIRS threshold criteria were met. Furthermore, additional logistic regression models were constructed with only two variables, where one was commonly CR (i.e., CR+PCT, CR+WBC, or CR+SIRS). These models showed an AUC >71%. When both predictors have values greater than their cutoff points, the probability of SAP was >50%. This further underscores the ability of CR plus any one of the other baseline parameters to predict SAP. We believe that a probability of 30% in the multivariate logistic regression model is a clinically relevant parameter to identify patients at risk of developing SAP.

Several AP severity scoring systems (APSSS) have been developed to assist in predicting patient clinical outcomes. When comparing existing APSSS statistically, the AUC for severity predicted by BISAP was 0.793, APACHE II was 0.836, Ranson score was 0.903 [20], and BALI score was 0.81 [21]. Our model had a comparable AUC of 0.824, with the added advantage of utilizing predictor variables, which can all be determined upon a patient’s presentation to a hospital. However, these other APSSS have deficiencies. The majority of these studies were based on small populations and retrospective data collected from cohorts that were not managed in a standardized fashion; this potentially introduces confounders and bias. Further, the relevance of APSSS is questioned if developed many years ago when management was different from current standards.

Many APSSS were developed without defining severity outcomes according to the RAC. The Glasgow criteria defined SAP as either fatal or associated with a complication that required a length of stay >20 days [22]. While the BISAP was derived and validated using mortality as the outcome [15], it has also been reported to correlate with organ failure [23-25] and SAP [26-31]. The most commonly recognized APSSS are cumbersome to calculate and require 48 hrs [32-34]. The APACHE II score was reported to best predict mortality [35], but has limited sensitivity to predict severity on admission [36], even in patients initially admitted to the ICU [37]. A simplified prognostic score was proposed, but it still requires 48 hrs [38]. Urea and glucose criteria at admission have been studied but not widely adopted [39,40]. The BALI score, composed of age and BUN, lactate dehydrogenase, and IL-6 levels, consistently predicted mortality from admission to 48 hrs, but cytokine measurement may not be practical [21]. Sensitivity for predicting SAP using the “early” warning score was low until after day two or three [41]. Some studies included the worst variables within the first 24 hrs, not necessarily at admission [15,42]. Utility of the Panc 3 score on admission, composed of BMI, HCT, and pleural effusion [43], may be limited as the latter two may require time. The Prediction Pancreatitis Severity (PPS) Score I uses five criteria for admission (age, BMI, CR, gender, and WBC) but requires a complicated calculation; the PPS II added CRP at 48 hrs to the calculation [44]. The Pancreatitis Outcome Prediction score was developed using retrospective data from patients admitted to ICUs [42]. Several of earlier APSSS have been modified to simplify the process and/or improve accuracy [22,45-48]. Some APSSS require CT imaging [49,50]. A contrast-enhanced CT scan is not recommended on admission unless the diagnosis is not clear and/or other complications are suspected [13], and it does not improve outcomes [51]. The revised Japanese APSSS recommends contrast-enhanced CT scanning to clarify prognosis in patients predicted to have SAP by laboratory and clinical criteria [48]. Some nonspecific physiology assessments are used as APSSS; however, they are not designed for AP [52,53]. Lastly, scoring systems such as BISAP and APACHE II utilize subjective criteria, such as altered mental status and the Glasgow coma scale, respectively, to predict severity, which again introduces confounders into datasets and analyses.

Thus, current methods for predicting AP severity have reached maximum utility, and there has been a call for new approaches with practical utility [7,8,54-59]. Existing APSSS can accurately predict a higher risk of mortality [15,41,49,60,61]. However, the goal should be to identify all patients at risk for even nonfatal SAP. Further, while most patients with AP are predicted to have a severe attack, only about half are ultimately graded as having a severe attack [10]. Conversely, AP can be fatal in even patients not predicted to have SAP [7,62-64]. The APACHE II [65], Ranson [32], Glasgow [66], and BISAP [7,67-70] are reported to have limited values in predicting SAP. For example, while patients with a BISAP score ≥3 are predicted to have increased mortality, one study reported that only just over half will ultimately be classified as having SAP [64]. Authors of a systemic analysis of BISAP studies claimed that, while it has a very good performance for predicting SAP, the small number of prospective studies and heterogeneity limits firm conclusions [71]. Importantly, BISAP has a low negative predictive value. Several studies have reported patients with fatal AP that had BISAP scores of ≤2 [24,63,64].

Our analysis of a prospective study registry is differentiated from previous assessments of potential individual biomarkers and various scoring systems. The data analyzed in this cohort were collected from a population of patients in which the EMM adhered to a standardized evidenced-based protocol. We used a computerized physician order set that guided early aggressive fluid resuscitation and early enteral feeding, as well as a patient navigator, to optimize adherence to APPG. These factors minimized confounders stemming from variable management. Lastly, we excluded variables that introduced subjectivity in interpretation (e.g., altered mental status). Our study identified variables that could be easily obtained and utilized clinically.

Other groups have used CART analysis to assess severity in AP. There are principle differences between these and our study. They were retrospective analyses of patients not managed by a standard EMM and did not use the RAC for the definition of SAP. The BISAP severity system was the first APSSS developed using CART analysis [15]. Though widely studied for the assessment of AP severity, BISAP was developed using mortality as the outcome. The other two studies used organ failure as a proxy for SAP. The CART analysis performed by Hong et al. identified three variables (BUN, pleural effusions, and calcium) as predictive for SAP [72]. They commented that SIRS might have been a significant variable with a larger sample size. Wang et al. identified five severity predictive variables (calcium, CR, age, partial thromboplastin time, and CT severity score) [73]. We performed a univariate analysis on 18 variables that have been investigated in previous studies. Using the RAC definition for SAP, our CART analysis was run on the variables found to be significant in the univariate analysis as well as variables that were considered to be clinically significant. A small change in the data can cause a large change in the structure of the CART model, resulting in instability. CART models also lack principled probabilistic frameworks like confidence intervals and odds ratios.

We used logistic regression as a robust strategy to develop a reliable clinical decision rule that could be used to classify patients by AP severity. There are numerous benefits of utilizing logistic regression modeling as an analytical tool as opposed to CART analysis. Logistic regression is a traditional statistics method, easier to implement and interpret, and very efficient to train. The sample size makes minimal modification of the coefficients, and it makes no assumptions about distributions of classes in feature space. Logistic regression also has minimal overfitting problems with small to moderate sample sizes. The logistic regression model not only provides a measure of how appropriate a predictor (coefficient size) but also its direction of association (positive or negative).

Our logistic regression analysis identified four baseline predictors: CR, WBC, PCT, and SIRS. A number of other studies have evaluated the importance of these variables for assessing the severity of AP. Increased serum CR during the first 48 hrs after admission was reported by Muddana et al. as associated with pancreatic necrosis [74]. In a study with a low prevalence of elevated CR (<5%), CR levels at any time during the first 48 hrs had low sensitivity but high specificity and negative predictive value for pancreatic necrosis [75]. There is potential for confounding effects (e.g., worsened CR after a contrast-enhanced CT scan and under- or over-fluid resuscitation), when CR is assessed as a biomarker during a period after admission (e.g., at 48 hrs). Others found that baseline CR was helpful in predicting mortality in patients with AP [76,77]. A large Italian study reported that CR >2.2 mg/dL within 24 hrs of admission was associated with both an increase in mortality and pancreatic necrosis [78]. Wan et al. reported that baseline CR >1.8 mg/dL was a useful predictor for AP severity with a 60% positive predictive value for persistent organ failure [79]. Our data suggest that a lower threshold for an elevated baseline CR is important.

The early APSSS models considered elevated WBC as a prognostic factor for SAP. A WBC >16K was associated with SAP in the Ranson criteria [32]. The threshold for WBC was similar (>15K) for SAP from the Glasgow criteria [22]. A prospective study from Turkey reported that leukocytosis and elevated CRP were associated with SAP [35]. The complicated PPS equation incorporates WBC in addition to CR, CRP, BMI, age, and gender [44]. A large prospective Polish study reported that WBC >13K was associated with mortality [29]. Analysis of our data suggests that a lower threshold (>11K) is predictive of SAP.

Early studies suggested that PCT, the 116 amino acid peptide precursor of calcitonin that is elevated early (3-24 hours) in severe inflammation and sepsis [80], can accurately discriminate patient risk for infected pancreatic necrosis [81,82]. Infected necrosis is of particular importance as it has the most influence on mortality (>30%) [83,84]. Our study found PCT to be the second most important variable in severity prediction. A small prospective study (N=75) reported that a baseline PCT >0.7 ng/mL did not accurately predict SAP [85]. A systemic analysis of existing scoring methods found that PCT was the best predictor for infected pancreatic necrosis [56]. There are two meta-analyses of prospective studies comparing serum PCT against recognized scoring systems to assess AP severity. Both reported no significant heterogeneity and the diagnostic utility to predict SAP and infected pancreatic necrosis was the highest for PCT >0.5 ng/ml [86,87]. One prospective study reported that elevated PCT at baseline and at 24 hrs correlated with SAP and performed better than both APACHE II and Ranson scores [88]. A recent prospective study reported that PCT >0.9 ng/mL was associated with a 51-fold risk of developing SAP [89]. Another recent prospective study found that PCT was comparable to BISAP and superior to other biomarkers for predicting severity, organ failure, and mortality in patients with AP [90].

Meeting SIRS criteria was an important variable in our analyses. Initiation of local inflammation associated with AP may lead to SIRS as a result of high circulating levels of proinflammatory cytokines that induce activated leukocytes, oxygen free radicals, ischemia-reperfusion, and organ injury [91-93]. Propagation of SIRS may result from hypoperfusion and intestinal ischemia that causes increased intestinal permeability and bacterial translocation [94]. Fluid sequestration, as a marker of SAP, is reported to be associated with SIRS criteria on admission [95]. A number of severity assessment systems have also used SIRS as a scoring component, including BISAP [15], Japanese severity scoring system [50], and PASS [96]. The Japanese severity scoring system, however, requires that more than two SIRS criteria are met [60]. One study of the early warning score (EWS) system reported that the EWS correlated with SIRS but that SIRS on admission was a better predictor of mortality [41].

Meeting SIRS criteria at baseline and persistent SIRS has been commonly studied in AP. Some prospective studies reported that baseline SIRS correlated with AP severity [97-99], while others did not [5]. A systemic review concluded that SIRS within 48 hrs was not an adequate predictor of persistent organ failure [56]. A large retrospective study (n=514) found that SIRS on day one was independently associated with moderate SAP after controlling for age, BUN, HCT, CR, and BMI [100]. An early prospective study of patients with predicted SAP (by APACHE II) reported a correlation with mortality among those patients meeting SIRS criteria on admission (72%) and those with early organ dysfunction (44%) [101]. Singh et al. reported a significant correlation between the number of SIRS criteria on admission and markers of severity, but persistent organ failure was considerably higher (22%) for those patients meeting four SIRS criteria [102]. The use of baseline SIRS criteria at admission may potentially lack predictive accuracy if either the time duration from symptom onset to admission is short (i.e., there has not been enough time to generate a cytokine response) or if anti-inflammatory sufficiently counteract proinflammatory mediators. For example, one study reported 8% mortality associated with SIRS on admission, but up to 25% mortality with persistent (within the first 48 hrs) SIRS, of which 36% did not have SIRS on admission [3]. Studies related to fluid resuscitation have reported improved outcomes using SIRS as a measure for early versus late fluid resuscitation [103] and Ringer’s lactate versus normal saline [104]. Thus, the use of SIRS in our analyses is strengthened due to the prospective standardized EMM design of our study.

The strengths of our study are derived from having a standardized cohort in which patients were all managed in a very deliberate, evidence-based manner. This minimized the confounders that are inevitably present in retrospective studies of cohorts that have been managed at the discretion of varied providers. Another strength of our study was the utilization of both logistic regression and CART analyses to identify the baseline variables most important in predicting AP severity, determine cutoffs, and create a decision tree model that physicians can implement easily in their clinical practice. Logistic regression as a traditional statistics method is easier to implement, interpret, and very efficient to train, whereas CART model calculations can be far more complex compared to other algorithms.

Our study is limited to the smaller population of patients with SAP in our cohort. This is perhaps explained by the standardized EMM by which all patients were treated. There is also perhaps some variability from symptom onset to admit as these data were not collected.

## Conclusions

Our severity of AP prediction (SAPP) model, namely, CR + WBC, PCT, and/or SIRS, will be based on easy-to-collect on admission, simple, and objective variables to predict severity. The next step is to validate these results in other patient populations that may not have been treated with a standardized EMM. Moreover, the SAPP could be used now to triage patients on admission to different EMM protocols and for proper disposition to ward or ICU care.
